# ABCD2 score has equivalent stroke risk prediction for anterior circulation TIA and posterior circulation TIA

**DOI:** 10.1038/s41598-023-41260-9

**Published:** 2023-08-26

**Authors:** Shuang Cao, Lu Zhao, Lulu Pei, Yuan Gao, Hui Fang, Kai Liu, Hao Liu, Shuxiang Yang, Shilei Sun, Jun Wu, Bo Song, Yuming Xu

**Affiliations:** 1 Department of Neurology, The First Affiliated Hospital of Zhengzhou University, No. 1 East Jianshe Road, Zhengzhou, 450052 Henan Province People’s Republic of China; 2 Department of Magnetic Resonance, The First Affiliated Hospital of Zhengzhou University, Zhengzhou, Henan Province China

**Keywords:** Neurology, Risk factors

## Abstract

Transient ischemic attack (TIA) was clinically divided into anterior circulation (AC) or posterior circulation (PC). Previous study reported that ABCD2 score could predict the stroke risk after AC-TIA but might have limitation for PC-TIA. We aimed to classify TIA depending on neuroimaging and assess the value of ABCD2 score for predicting stroke risk in different territories. Research data was from TIA database of the First Affiliated Hospital of Zhengzhou University. TIA patients with acute infarction on diffuse weighted imaging [that is, transient symptoms with infarction (TSI)] were divided into anterior and posterior circulation groups according to the location of infarction. The outcome was recurrent stroke within 7 and 90 days. The predictive power of ABCD2 score was determined using area under receiver operator characteristic curve (AUC) analyses. Overall, 382 AC-TSI and 112 PC-TSI patients were included. There were 38 (9.9%) AC-TSI patients and 11(9.8%) PC-TSI patients who had recurrent stroke at 7 days, and 66 (17.3%) AC-TSI patients and 19 (17.0%) PC-TSI patients who had recurrent stroke within 90 days. At 7 days, the AUC for ABCD2 score was 0.637 (95% confidence interval CI 0.554–0.720) in anterior circulation and 0.683 (95% CI 0.522–0.845) in posterior circulation. The C statistics for ABCD2 score in the two groups were not statistically significant (Z =  − 0.499; *P* = 0.62). Similar result was found when the outcome time-point was set at 90 days. ABCD2 score could predict the short-term risk of recurrent stroke after AC-TSI and PC-TSI, and had similar predictive abilities for AC-TSI and PC-TSI.

## Introduction

Patients who had transient ischemic attacks (TIAs) carried high early risks of subsequent stroke^1–3^, regardless of anterior circulation TIA (AC-TIA) or posterior circulation TIA (PC-TIA). In clinical practice, TIAs were usually classified into the different circulations by typical symptoms^4^. The effectiveness of early interventions to prevent stroke after TIA has led to the need for simple screening methods to identify patients with acute stroke and TIA^5^. The TIAregistry.org project, a prospective international registry of patients who had a recent TIA or minor ischemic stroke (MIS), recommended the ABCD2 score as an effective score for stratifying risk of patients with TIA or MIS^6^, and another study also indicated that ABCD2 score was well validated in Chinese population^7^.

ABCD2 score might have different predictive performance in different circulations, such as motor deficits and speech disturbance, the component of ABCD2 score, were mostly presented in anterior circulation^8^. Previous studies classified TIA according to typical clinical symptoms and reported that ABCD2 score could predict the risk of stroke after AC-TIA, but might have limitation for PC-TIA^4^. However, ischemic events in the anterior circulation and posterior circulation can produce same symptoms, identification of the territories on the basis of clinical symptoms and signs might lead to misclassification^9^. Moreover, several recent studies reported that up to 21–68% TIA patients had acute infarction on diffuse weighted imaging (DWI), which was defined as transient symptoms with infarction (TSI)^10^, and had a higher risk of stroke than those without acute infarction on DWI^11,12^. If these patients were distinguished into anterior circulation or posterior circulation by neuroimaging, could they be classified more accurately and take proper therapies in time?

Therefore, we divided TSI patients into anterior circulation TSI (AC-TSI) and posterior circulation TSI (PC-TSI) according to the location of infarction in this study, and we aimed to assess the value of the ABCD2 score for predicting stroke risk after either AC-TSI or PC-TSI.

## Materials and methods

### Study population

Patients included in this study were from the TIA database of the first affiliated hospital of Zhengzhou University, which is a prospective hospital–based cohort study. The detailed, rationale, and basic description of this database have been published previously^13,14^. TIA was diagnosed based on World Health Organization (WHO) diagnostic criteria, which defined a TIA as an acute loss of focal cerebral or ocular function lasting less than 24 h attributed to embolic or thrombotic vascular diseases^15^. Enrolled patients attended hospital within 7 days after the onset of symptoms. Patients who had incomplete ABCD2 score and infarctions occurring concurrently in both the anterior and posterior circulation were excluded. All subjects gave their informed consent for inclusion before they participated in the study. The study was conducted in accordance with the Declaration of Helsinki, and the protocol was approved by the Ethics Committee of the First Affiliated Hospital of Zhengzhou University.

### Data collection

Trained physicians recorded all information of patients by face-to-face interviews, including age, gender, clinical features, medical history, imaging features, risk predictive scores and follow-up information. DWI was performed as a routine piece of diagnostic evaluation in all TIA patients who did not have contraindication to magnetic resonance imaging (MRI) within 7 days of the index TIA. The index TIA was defined as the most recently preceding assessment by a stroke specialist.

All patients enrolled were evaluated with the ABCD2 score by neurologists at the time of admission to hospital. The score was based on five factors: age ≥ 60 years 1point; systolic blood pressure ≥ 140 mmHg or diastolic blood pressure ≥ 90 mmHg 1point; clinical features: unilateral weakness (2 points), speech impairment without weakness (1 point); duration ≥ 60 min (2 points) or 10–59 min (1 point) and diabetes mellitus (1 point)^16^.

### Follow-up and outcome

The patients enrolled in the TIA database were followed up at 2th and 7th days at admission by face-to-face interviews, the well-trained neurologists contacted the patients by telephone at 3, 6, 9 and 12 months after discharge and recorded the events and exact time of stroke recurrence that occurred during follow-up. The outcome was judged by distinctive clinical symptoms and confirmed by neuroimaging examination. All patients suspected of stroke were followed up via face-to-face interview and recommended to undergo brain computed tomography (CT) or magnetic resonance imaging (MRI) to confirm end point events. If some patients visited other hospitals, we would verified the outcome by checking medical records and relative materials which were obtained from the provincial network system. The end points for this study were stroke within 7 and 90 days. Stroke included ischemic and hemorrhagic strokes. Ischemic stroke was defined as a new focal neurological deficit lasting for ≥ 24 h, with clinical or imaging evidence of infarction and not ascribed to a nonischemic cause^17^. Hemorrhagic stroke was defined as acute extravasation of blood into the subarachnoid space or brain parenchyma with associated neurological symptoms^17^.

### Statistical analysis

Participants were assigned to the anterior circulation and posterior circulation group according to the infarction location in DWI sequence. Categorical variables were compared using the Pearson chi-square test, or Fisher’s exact test if numbers in the 2 × 2 table were too low. Continuous variables were compared between groups using independent sample t-test or Mann–Whitney U-test. The risk of each ABCD2 score was calculated and was test by Cochran-Armitage trend. The predictive ability of the ABCD2 scores was evaluated by the receiver operating characteristic curves (ROCs). Discrimination was assessed by calculating the area under the receiver operating characteristic curve (AUC). Calibration was assessed by performing the Hosmer–Lemeshow goodness of fit test. Logistic regression analysis was applied to identify independent predictors associated with recurrent stroke. Associations were presented as odd ratio (OR) with corresponding 95% confidence interval (CI). Statistical significance was established at the *P* < 0.05 level. All statistical analyses were performed using SPSS software for Windows (version 25.0; SPSS).

### Ethics approval and consent to participate

The study was conducted according to the guidelines of the Declaration of Helsinki, and approved by the Ethics Committee of the First Affiliated Hospital of Zhengzhou University (Prot. 45/2010, October 2010). Informed consent was obtained from all subjects involved in the study.

## Results

A series of 1652 consecutive patients with a diagnosis of TIA were prospectively enrolled from October 2010 to August 2017. There were 554 patients who had infarctions on DWI, which were called TSI patients. A total of 57 patients had infarctions occurring concurrently in both the anterior and posterior circulation, and 3 patients lost to follow-up at 90 days. Ultimately, 494 eligible patients were included in the final analysis. Table S1 showed the baseline characteristics of included and excluded patients, and there was no difference in the two groups except age.

Of the 494 eligible patients, 382 (77.3%) were AC-TSI patients and 112 (22.7%) were PC-TSI patients. Table 1 showed the demographic characteristics, risk factors and treatments of the two groups of patients. There were no statistical differences in this risk factors and secondary prevention treatment except history of hypertension, which had an incidence of 64.3% in the PC-TSI group compared with 53.7% in the AC-TSI group (*P* = 0.046). Moreover, the patients with PC-TSI were more likely to be men (74.1% vs. 63.4%, *P* = 0.035) and had a lower ABCD2 score (median, 3 vs. 4, *P* = 0.031) compared with patients with AC-TSI. We further analyzed the difference of each component of ABCD2 score between the two groups. Patients with PC-TSI were older (≥ 60 years, 53.6% vs. 42.1%, *P* = 0.032) and had higher blood pressure (80.4% vs. 69.6%, *P* = 0.026). Speech disturbance (7.1% vs. 13.9%) and unilateral weakness (47.3% vs. 70.4%, *P* < 0.001) occurred more often in the anterior territory than in posterior territory. The duration of symptoms and diabetes mellitus in two territories was not statistically significant.

**Table 1:** Baseline characteristics of patients with TSI.

	Anterior circulation (n = 382)	Posterior circulation (n = 112)	*P* value
Age ≥ 60 y	161 (42.1%)	60 (53.6%)	0.032
Male	242 (63.4%)	83 (74.1%)	0.035
Current smoking	135 (35.5%)	39 (35.1%)	0.940
Medical history			
Hypertension	205 (53.7%)	72 (64.3%)	0.046
Diabetes	74 (19.4%)	19 (17%)	0.567
Dyslipidemia	74 (19.4%)	28 (25%)	0.196
Coronary heart disease	40 (10.5%)	17 (15.2%)	0.170
Atrial fibrillation	11 (2.9%)	2 (1.8%)	0.764
History of stroke	92 (24.1%)	26 (23.2%)	0.849
SBP ≥ 140 mmHg or DBP ≥ 90 mmHg	266 (69.6%)	90 (80.4%)	0.026
Clinical symptom			
Speech disturbance without weakness	53 (13.9%)	8 (7.1%)	< 0.001
Unilateral weakness	269 (70.4%)	53 (47.3%)	
Duration			
< 10 min	134 (35.1%)	42 (37.5%)	0.604
10–59 min	170 (44.5%)	44 (39.3%)	
≥ 60 min	78 (20.4%)	26 (23.2%)	
ABCD2 score	4 (3–5)	3 (2–4)	0.031
Discharge treatment			
Antiplatelet agents	363 (95%)	110 (98.2%)	0.228
Anticoagulant	18 (4.7%)	4 (3.6%)	0.799
Lipid-lowering agents	354 (92.7%)	110 (98.2%)	0.053
Antihypertension agents	125 (32.7%)	38 (33.9%)	0.811
Hypoglycemic agents	80 (20.9%)	22 (19.6%)	0.765

*TSI* transient symptom with infarction, *SBP* systolic blood pressure, *DBP* diastolic blood pressure.

**Figure 1:**
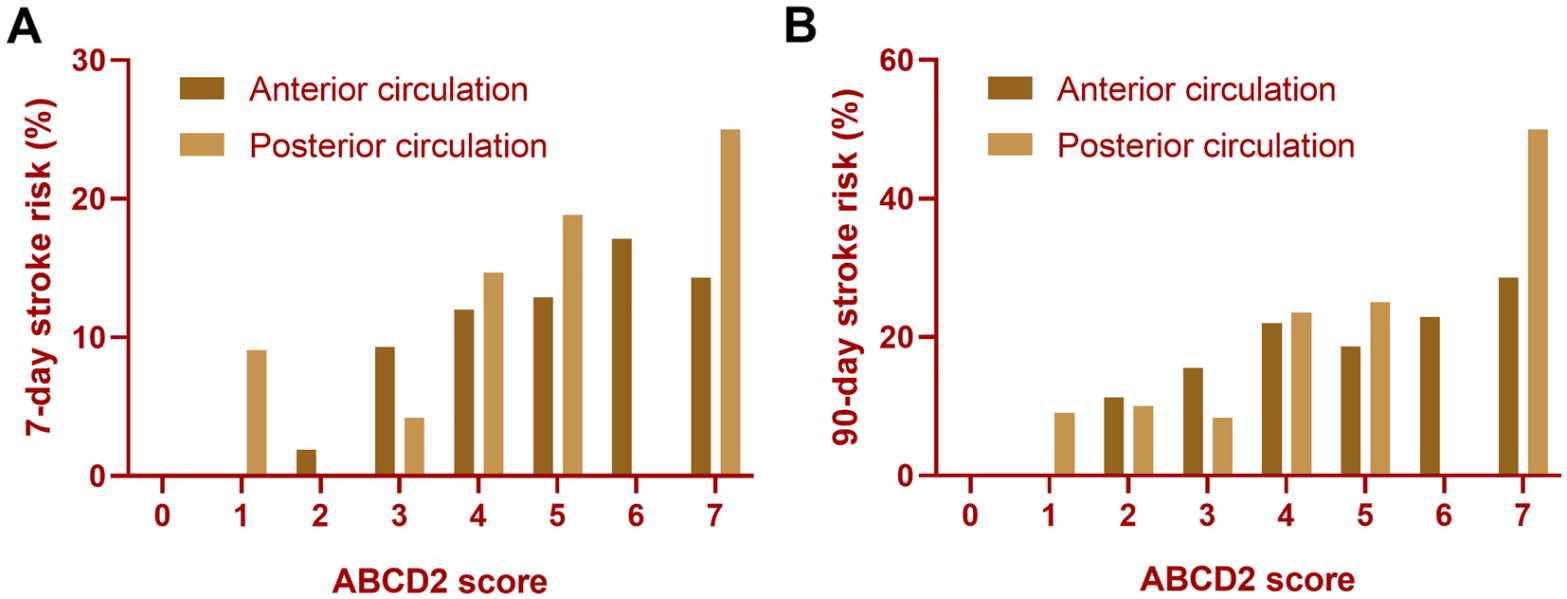
Incidence of 7-day and 90-day stroke stratified according to the ABCD2 score in the anterior or posterior circulation TSI. Cochran-Armitage trend test showed a linear trend existed on 7 days (**A**, AC-TSI, Z =  − 2.8525, *P* = 0.004; PC-TSI, Z =  − 2.1457, *P* = 0.032) and 90 days (**B**, AC-TSI, Z =  − 2.5415, *P* = 0.011; PC-TSI, Z =  − 2.2013, *P* = 0.028).

**Figure 2:**
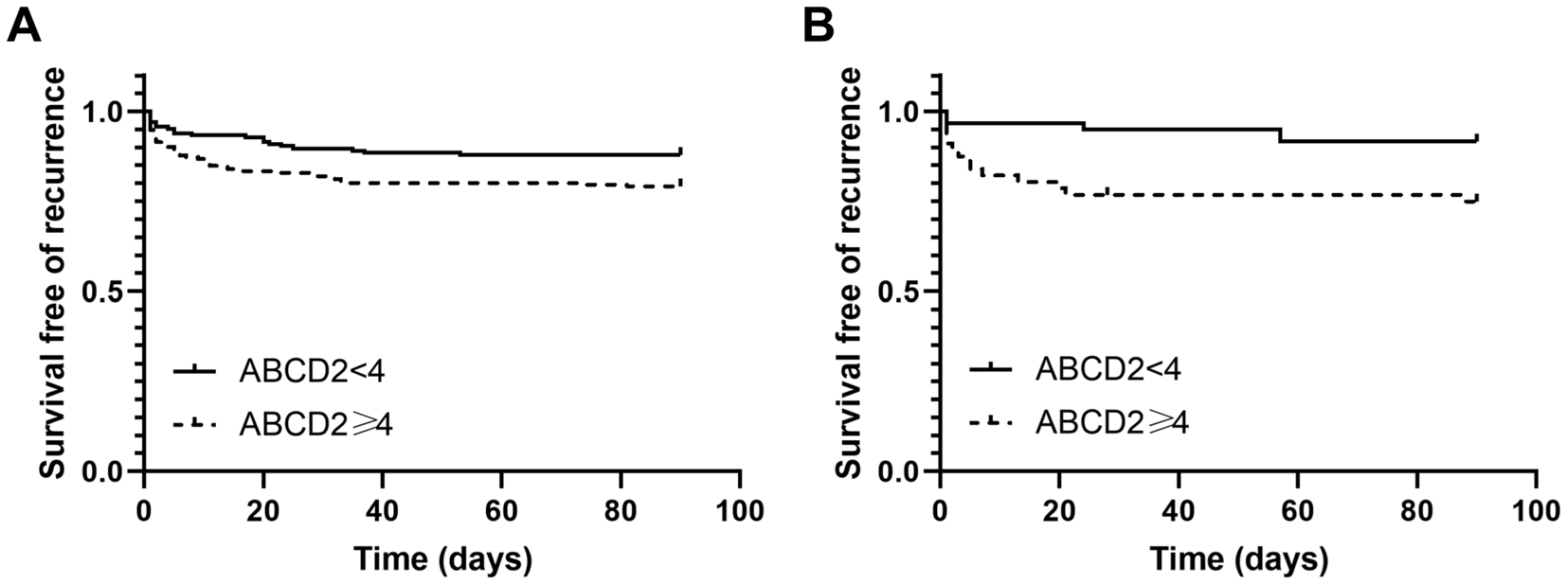
Kaplan–Meier curves of patients stratified according to the ABCD2 score. ABCD2 score was associated with elevated risk of stroke in anterior circulation (**A**, log rank *P* = 0.020) and posterior circulation (**B**, log rank *P* = 0.020).

At 7-day follow-up, there were 38 (9.9%) patients who had recurrent stroke in AC-TSI group and 11 (9.8%) patients in PC-TSI group, while at 90-day follow-up, 66 (17.3%) AC-TSI patients and 19 (17.0%) PC-TSI patients had recurrent stroke. Figure 1 showed the stroke risk according to different ABCD2 score in patients with AC-TSI and patients with PC-TSI, which illustrated an overall increase in the rate of stroke with increasing ABCD2 score. A linear trend for occurrence rates was observed with the Cochran-Armitage trend test on 7 days (AC-TSI, Z =  − 2.8525, *P* = 0.004; PC-TSI, Z =  − 2.1457, *P* = 0.032) and 90 days (AC-TSI, Z =  − 2.5415, *P* = 0.011; PC-TSI, Z =  − 2.2013, *P* = 0.028). When ABCD2 score was treated as a categorical variable, Kaplan–Meier analysis showed that the higher ABCD2 score was associated with elevated risk of recurrent stroke in anterior circulation (log rank *P* = 0.020) and posterior circulation (log rank *P* = 0.020, Fig. 2).

**Figure 3:**
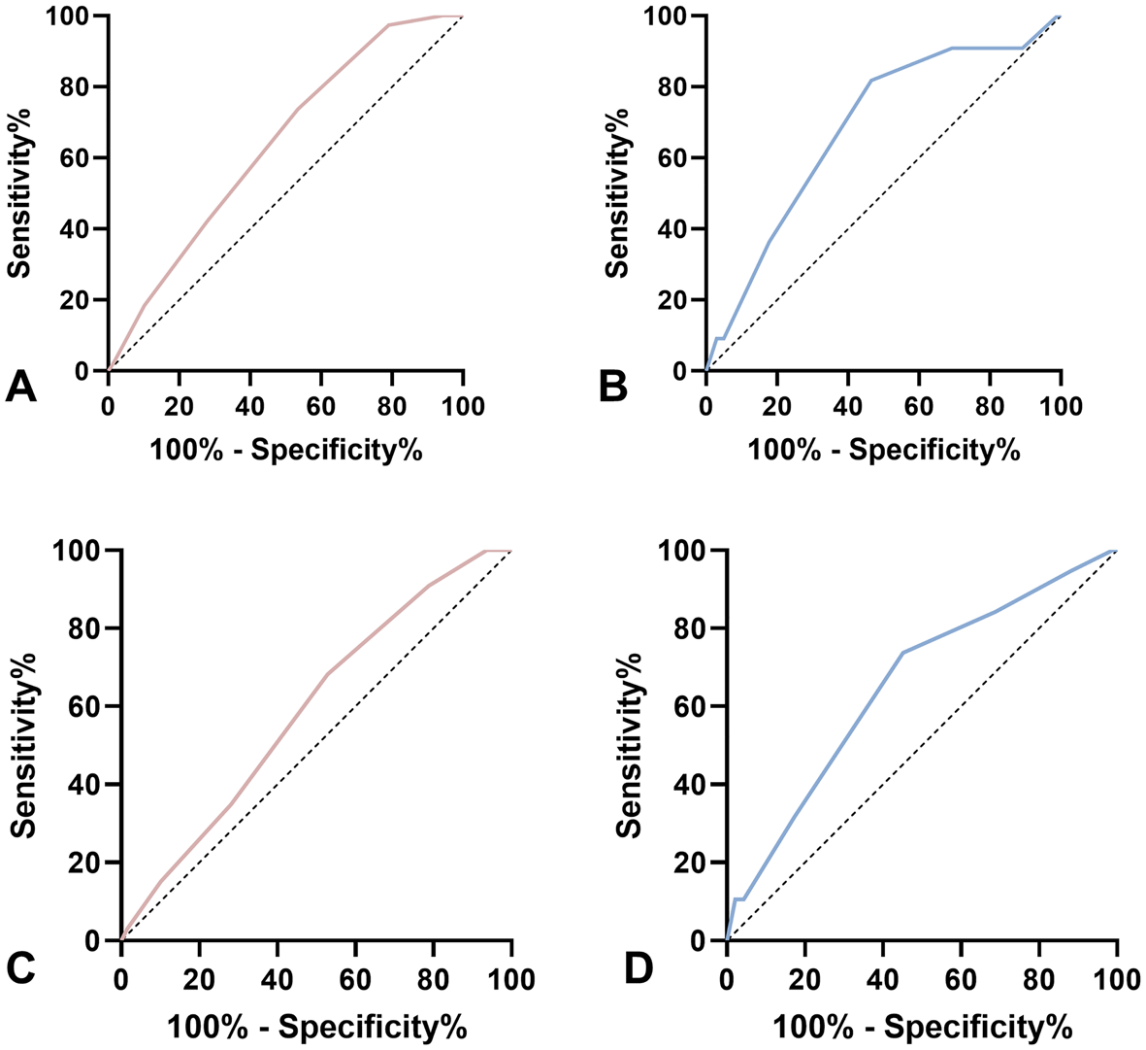
Receiver-operating characteristic curves as a predictive value of the ABCD2 score in different territories TSI patients. The area under the curve (AUC) for ABCD2 score was 0.637 (**A**) in anterior circulation and 0.683 (**B**) in posterior circulation at 7 days. The area under the curve (AUC) for ABCD2 score was 0.592 (**C**) in anterior circulation and 0.653 (**D**) in posterior circulation at 90 days.

Figure 3 showed the ROC curve for predicting risk of recurrent stroke in patients with AC-TSI and PC-TSI. At 7 days, the AUC of ABCD2 score was 0.637 (95% confidence interval CI 0.554–0.720) in anterior circulation and 0.683 (95% CI 0.522–0.845) in posterior circulation. The C statistics for ABCD2 score in the two groups were not statistically significantly different (Z =  − 0.499; *P* = 0.62). The goodness of fit test showed that the Hosmer–Lemeshow χ^2^ of ABCD2 score was 3.172 (*P* = 0.530) in anterior circulation and 3.836 (*P* = 0.429) in posterior circulation. At 90 days, the AUC of ABCD2 score was 0.592 (95% CI 0.541–0.642) in anterior circulation and 0.653 (95% CI 0.557–0.741) in posterior circulation. Similar result was found when the outcome timepoint was set at 90 days (*Z* =  − 0.800, *P* = 0.42). The goodness of fit test showed that the Hosmer–Lemeshow χ^2^ of ABCD2 score was 3.387 (*P* = 0.495) in anterior circulation and 1.211 (*P* = 0.876) in posterior circulation.

The multivariable logistic regression analysis was performed to determine independent risk factors associated with recurrent stroke (Table 2). Notably, unilateral weakness (adjusted odd ratio [OR], 4.873, 95% CI 1.460–16.258) was associated with recurrent stroke after AC-TSI. However, for PC-TSI patients, duration more than 10 min (adjusted OR, 8.732, 95% CI 2.124–35.897) was found to be associated with a risk of recurrent stroke within 90 days.

**Table 2:** Multivariable logistic regression analysis of individual risk factors for stroke events after TSI.

	Anterior circulation			Posterior circulation		
	Adjusted^a^ OR	95%CI	*P* value	Adjusted^a^ OR	95%CI	*P* value
Age ≥ 60	1.226	0.704–2.134	0.472	2.299	0.705–7.498	0.167
SBP ≥ 140 mmHg or DBP ≥ 90 mmHg	1.978	1.006–3.889	0.048	8.803	0.989–78.360	0.051
Clinical symptom						
Speech disturbance without weakness	3.753	0.949–14.831	0.059	0.877	0.076–10.074	0.916
Unilateral weakness	4.873	1.46–16.258	0.010	2.201	0.665–7.282	0.196
Duration						
< 10 min	–	–	Ref	–	–	Ref
10–59 min	0.936	0.502–1.744	0.835	8.732	2.124–35.897	0.003
≥ 60 min	1.058	0.501–2.233	0.882	1.386	0.203–9.447	0.739
Diabetes mellitus	0.782	0.383–1.595	0.499	0.921	0.233–3.634	0.906

^a^Adjusted for age, blood pressure at admission, clinical symptoms, duration of symptoms and diabetes mellitus.

*TSI* transient symptoms with infarction.

## Discussion

In this present study, we divided tissue-positive TIA into anterior circulation and posterior circulation according to infarction location on DWI, and we found that ABCD2 score could predict the short-term risk of recurrent stroke after anterior circulation TSI and posterior circulation TSI, and had equivalent predictive abilities in the two territories.

In clinical practice, A fifth of all TIAs and ischemic strokes were in the territory of the posterior circulation^18^. In our study, the AC-TSI accounted for 77.3% and PC-TSI account for 22.7%, which was similar to the previous studies. The incidence of recurrent stroke was 9.9% at 7 days and 17.2% at 90 days in our study, which were higher than those documented in previous studies^19,20^. Differences in ethnic factors, demographics and hospital-related factors may explain the variations between studies. There has not been a definite conclusion about the comparison of the risk of subsequent stroke in patients with anterior and posterior TIA. A meta-analysis found that patients presenting with TIA or minor stroke in posterior circulation were at increased risk for subsequent stroke in the acute period and at reduced risk for subsequent stroke in the chronic period^21^. However, several studies had conflicting results^4,22^. Possible reason for the discrepancy may be that studies used the different definitions when classifying the neurological deficits. As we all know, there have not been reliable clinical criteria for PC-TIA. Moreover, in consideration of some symptoms and signs could be similar in anterior circulation and posterior circulation, so it was difficult to recognize corresponding territories according to diverse symptoms^23^. Neuroimaging was vital to ensure accurate localization. Our study showed that anterior circulation and posterior circulation TSI had similar incidence of recurrent stroke at 7 and 90 days. And more researches were needed to verify the result.

The ABCD2 score has been recommended for use in international guidelines to improve early stroke risk stratification after TIA^24,25^, and help primary care and emergency clinicians to rapidly identify high-risk TIA patients^16,26^. The clinical manifestation of TIA patients was diverse usually but differences exist between anterior circulation and posterior circulation. The symptoms of AC-TIA were mostly motor deficits, sense deficits and speech disturbance, and the symptoms of the PC-TIA were more variable than AC-TIA. The New England Medical Center posterior circulation registry revealed that the common symptoms in posterior circulation were dizziness, gait ataxia, dysarthria, and diplopia^23^. Moreover, the duration of symptoms was different between these territories. These dissimilarities indicated that ABCD2 score could have different abilities in predicting the risk of recurrent stroke in AC-TIA and PC-TIA. Recent study revealed that tissue-positive events with low ABCD2 scores and tissue-negative events with high ABCD2 scores had similar stroke risks, and ABCD2 score had a validity in predicting stroke risk in tissue-positive TIA^27^. In our study, the AUC for predicting recurrent stroke was between 0.592 and 0.683. Other scores such as ABCD3I score and RRE score had high predictive value of stroke risk but they need to more neuroimaging examination to evaluate patients exactly^14,28^. But in the emergency sitting, the ABCD2 score was more available. The ABCD2 score is used widely around the world and it has been recommended for use in international guidelines to improve early stroke risk stratification after TIA. Besides, the result of this study showed that ABCD2 score had the ability of discrimination and calibration in predicting stroke risk, and ABCD2 score had equivalent predictive values in anterior circulation and posterior circulation TSI patients.

However, the previous study reported that ABCD2 score could predict the short-term risk of stroke after AC-TIA, but might have limitation for PC-TIA^4^. And in another study, the ABCD2 score was less effective in the diagnosis and identification of high-risk posterior circulation stroke and TIA^29^. The reason why ABCD2 score could not predict stroke risk after PC-TIA might be that previous studies differentiate AC-TIA and PC-TIA according to typical clinical symptoms. However, A substantial proportion of PC-TIA patients might not be accurately classified only by symptoms or signs because they lack typical clinical feature. One previous study observed that approximately 10–20% of patients with a diagnosis of presumed anterior circulation infarction actually had a posterior circulation infarction^30^. Inaccurate localization would occur commonly if clinicians relied on the clinical neurological deficits alone to differentiate AC and PC. In our study, tissue-positive TIA was divided AC-TSI and PC-TSI according to infarction location, and the result showed the ABCD2 score had a predictive validity in the anterior and posterior circulation TSI. This result validated that imaging examination could ensure accurate localization and help to make more reliable classification.

Our study was the first time to divided TIA patients into anterior circulation and posterior circulation based on the location of infarction on DWI, and proved the ability of ABCD2 score in predicting recurrent stroke. However, there were some limitations in this study. Firstly, the sample of this study was small, and the patients were enrolled from a single-center hospital, there might have been selection bias. Secondly, the results of this study cannot be generalized to all TIA patients because we excluded patients with DWI–negative TIA and patients with infarction location occurring concurrently in both anterior and posterior circulation. Therefore, the results should be interpreted with caution.

## Conclusion

In conclusion, we divided tissue-positive TIA patients into anterior circulation and posterior circulation according to location of infarction, and we found that the ABCD2 score could predict the short-term risk of recurrent stroke after anterior circulation TSI and posterior circulation TSI, and had equivalent predictive abilities in the two territories.

### Supplementary Information

Supplementary Table S1.

## Data Availability

The datasets used and/or analyzed during the current study are available from the corresponding author on reasonable request.
