# Supplementary material for: ABCD2 score has equivalent stroke risk prediction for anterior circulation TIA and posterior circulation TIA
Source: Sci Rep. 2023 Aug 26;13:13993. doi: 10.1038/s41598-023-41260-9 (PMC10460395; doi:10.1038/s41598-023-41260-9)
Supplement: Supplementary file 1 — Supplementary Table S1. [file 41598_2023_41260_MOESM1_ESM.docx]

Table S1 Baseline characteristics of excluded and included patients.

|  | Excluded (n=60) | Included (n=494) | *P* value |
| --- | --- | --- | --- |
| Age | 61.58±13.74 | 56.93±12.54 | 0.009 |
| Male | 34(59.6%) | 326(65.6%) | 0.373 |
| Current smoking | 17(29.8%) | 174(35.2%) | 0.417 |
| Medical history | | | |
| Hypertension | 34(59.6%) | 278(55.9%) | 0.592 |
| Diabetes | 15(26.3%) | 94(18.9%) | 0.183 |
| Dyslipidemia | 6(10.5%) | 103(20.7%) | 0.067 |
| Coronary heart disease | 9(15.8%) | 57(11.5%) | 0.340 |
| Atrial fibrillation | 3(5.3%) | 13(2.6%) | 0.476 |
| History of stroke | 11(19.3%) | 118(23.7%) | 0.452 |
| ABCD2 score | 4(3-5) | 4(3-5) | 0.517 |
| Discharge treatment | | | |
| Antiplatelet agents | 54(94.7%) | 475(95.6%) | 0.773 |
| Anticoagulant | 3(5.3%) | 22(4.4%) | 0.773 |
| Lipid-lowering agents | 55(96.5%) | 466(93.8%) | 0.597 |
| Antihypertension agents | 17(29.8%) | 164(33%) | 0.628 |
| Hypoglycemic agents | 14(24.6%) | 102(20.5%) | 0.478 |
